# Oxidative Stress: Implications for the Development of Diabetic Retinopathy and Antioxidant Therapeutic Perspectives

**DOI:** 10.1155/2014/752387

**Published:** 2014-08-10

**Authors:** Ying Wu, Luosheng Tang, Baihua Chen

**Affiliations:** Department of Ophthalmology, Second Xiangya Hospital, Central South University, 139 Middle Renmin Road, Changsha 410011, China

## Abstract

In recent decades, localized tissue oxidative stress has been implicated as a key component in the development of diabetic retinopathy (DR). Increasing evidence shows that oxidative stress caused by diabetes-induced metabolic abnormalities is the most common mechanism associated with the pathogenesis of DR for both type 1 and type 2 diabetes. Increase in intracellular reactive oxygen species (ROS) concentrations results in the activation of several mechanisms involved in the pathogenesis of DR. In particular, damage or dysfunction caused by oxidative stress still persists even after glycemia has been normalized. Despite considerable evidence showing the beneficial effects of antioxidants in preventing the development of retinopathy, results from large-scale clinical trials on classic antioxidants are somewhat ambiguous. Scavenging reactive radicals may not be the most ideal antioxidant strategy in DR. Advances in understanding the function of ROS in the development of DR can lead to the development of new therapeutic strategies based on the mechanisms of ROS generation and scavenging. Increasing amounts of data have demonstrated the promising prospect of antioxidant therapy and its beneficial effects in vision protection. Therefore, new strategies that utilize antioxidants as additive therapy should be implemented in the treatment of DR.

## 1. Introduction

Diabetes mellitus (DM) is a lifelong progressive and the most common metabolic disease that has become the epidemic of the 21st century. Approximately 347 million people were diagnosed with diabetes in 2011 worldwide [1]. The World Health Organization predicts that diabetes will be the seventh leading cause of death in 2030 [2]. Diabetic retinopathy (DR), one of the microvascular complications in diabetes, is the major cause of blindness in adults. DR is characterized by gradual and progressive alterations in the retinal microvasculature. Damages to neurons and glia also occur during the course of DR. Individuals with diabetes, regardless of whether they are afflicted with type 1 or type 2, are all at risk of developing retinopathy. The longer a patient has diabetes, the higher the risk of developing DR is. Approximately 25% of patients with type 1 diabetes have been shown to have retinal damage, and the incidence increased to 60% after 5 years and 80% after 10 years to 15 years of affliction. Type 2 diabetes accounts for the higher prevalence of DR [3]. Systemic medication of tight control of glucose, blood pressure, and lipids can reduce the risk of developing DR. However, systemic mediation is hard to achieve clinically. The present standard therapeutic drug for DR is rare, and current management of DR is exclusively focused on vascular changes. Despite extensive research in the field, cellular and molecular bases of DR remain partially elucidated. Thus, further investigation of the mechanisms on how diabetes affects retina is necessary to develop new therapeutic treatments for DR.

Increasing evidence emphasizes the critical involvement of elevated oxidative stress in the pathogenesis of diabetes and its complications. The retina is particularly susceptible to oxidative stress because of high energy demands and exposure to light [3]. A number of interconnecting biochemical mechanisms that contribute to the pathogenesis of DR have been identified, including inflammation, the polyol pathway, accumulation of advanced glycation end products (AGEs), the flux of hexosamine pathway, and protein kinase C (PKC) activation. All of these mechanisms appear to be associated with mitochondrial overproduction of reactive oxygen species (ROS) [4]. In obesity and dyslipidemia, DR appears to be also associated with oxidation of fatty acids, resulting in increased production of ROS by nicotinamide adenine dinucleotide phosphate (NADPH) oxidase. Numerous drugs have been developed based on current understanding of oxidative stress in biochemical and pathophysiological aspects of DR. Given the many well-established antioxidants that have been used in DR pharmacotherapy, results from clinical trials involving antioxidant supplementation seem ambiguous. New mechanism-based therapeutic strategies have been explored and have promising potential. The present study discussed the involvement of oxidative stress in the pathogenesis of DR. Recent clinical and experimental progress in the development of pharmacotherapy for DR was also summarized.

## 2. Pathogenesis of DR

All forms of diabetes are characterized by hyperglycemia. The mainstay of diabetes treatment used to be blood glucose control to prevent or delay the development of various diabetic complications, including DR. Results of the land mark Diabetes Control and Complications Trail (DCCT) [5] and its follow-up study, the Epidemiology of Diabetes Interventions and Complications Study (EDIC) [6], found that intensified glycemic control reduces the occurrence and severity of diabetic complications. Seminal studies were conducted to confirm the importance of optimizing glycemic control in type 2 diabetes through the UK Prospective Diabetes Study (UKPDS) [7] and the Steno-2 study [8]. These large-scale prospective studies highlight the importance of hyperglycemia in the pathogenesis of diabetes and its complications. A number of hyperglycemia-induced metabolic abnormalities contribute to the pathogenesis of DR, including oxidative stress, inflammation, the polyol pathway, accumulation of AGEs, and PKC activation [9]. These clinical trials also show that even with careful glycemic control, complications, including retinopathy, nephropathy, vascular and cardiac conditions, and neuropathy, still affect many patients with diabetes. These data revealed that stressors of diabetic complications continue beyond the point when glycemic control has been achieved. In metabolic syndrome (MS), prolonged impaired glucose tolerance and insulin resistance occur, eventually leading to type 2 diabetes; hyperglycemia does not develop for a long time. In patients or experimental animals suffering from type 2 diabetes, hyperglycemia is the major determinant factor in not only the development and progression of diabetes and its complications, but also insulin resistance, glucose intolerance, obesity, and oxidative stress [10]. Epidemiological and prospective data suggest that DR is a multifactorial disease. In addition to hyperglycemia, other systemic factors, such as hypertension, dyslipidemia, and genetic factors, are also involved in the process.

In the past decades, oxidative stress-related mechanisms have been implicated in the pathology of various diseases. Oxidative stress is defined as a disturbance in the balance between the production of high reactive molecules (free radicals) and the biological system's ability to remove harmful effects through neutralization by antioxidants. Free radicals are highly reactive molecules that participate in important signaling mechanisms and cell homeostasis, which are essential for many physiological functions, and also act as second messengers. Oxidative stress caused by the oxidants of biological macromolecules, including proteins, lipids, carbohydrates, and DNA, results in the disruption in cellular homeostasis and generation of other reactive molecules that create further damage. The significance of oxidative stress and mechanisms in the pathology and progress of DM and its complications have been investigated by several groups, and a huge body of literature exists regarding this area of research.

### 2.1. Hyperglycemia and Oxidative Stress

The majority of publications discuss that the mechanisms underlying hyperglycemia-induced diabetic tissue damage in DM develop through the following four major mechanisms: (1) increased flux of the polyol pathway; (2) PKC activation; (3) increased intracellular production of AGEs; and (4) overactivation of the hexosamine pathway [9]. Some evidence indicated that all hyperglycemia-induced mechanisms are activated by a single upstream event, the mitochondrial overproduction of the ROS [4]. Meanwhile, results of studies in which only one of the pathways previously mentioned is blocked have been disappointing, [11–13] which leads to the hypothesis that overproduction of ROS and decreased efficiency of antioxidant defenses are a process that starts at a very early stage and eventually worsens over the course of the disease [4].

#### 2.1.1. Increased Flux of the Polyol Pathway

Increased intracellular glucose flux due to hyperglycemia activates the polyol pathway, which is also called the sorbitol-aldose reductase pathway. Aldose reductase (AR), the rate-limiting enzyme in the pathway, reduces unused glucose to sorbitol. The reaction oxidizes NADPH to NADP+. Sorbitol dehydrogenase can then oxidize sorbitol to fructose, which produces NADH from NAD+. In the hyperglycemic state, the affinity of AR for intracellular glucose rises, resulting in decreased NADPH levels. NADPH acts to regenerate glutathione (GSH) from oxidized glutathione (GSSG), whereas NADPH deficiency will also cause glutathione deficiency. GSH is an important scavenger of ROS; the decrease in GSH can induce or exacerbate oxidative stress [14]. NADPH also acts to promote the production of nitric oxide (NO), one of the most important vasodilators in blood vessels. Therefore, decreased levels of NADPH are responsible for the ROS accumulation and oxidative stress damage. Moreover, increasing the intracellular ratio of NADH/NAD+ leads to inhibition of glyceraldehyde 3-phosphate dehydrogenase (GAPDH), which, in turn, increases intracellular concentrations of triose phosphate. Increased concentrations of triose phosphate induce the formation of methylglyoxal, a precursor of diacylglycerol (DAG) and AGEs, thereby leading to the activation of the AGE and PKC pathways [15].

#### 2.1.2. Activation of PKC

PKC refers to a family of serine/threonine protein kinases that are grouped based on their activation mechanisms. PKC enzymes are activated by signals, such as increases in the concentration of DAG or calcium ions (Ca^2+^). Hyperglycemia activates PKC, which stimulates the formation of DAG. Increased ROS because of hyperglycemia inhibits activity of the glycolytic enzyme GAPDH, resulting in increased intracellular levels of the DAG precursor triose phosphate, which stimulates the synthesis of DAG from glucose via triose phosphate. PKC activation also contributes to ROS production and oxidative stress by increasing the activity of NADPH oxidases (Nox) [16]. Other effects induced by PKC activation include inhibition of endothelial nitric oxide synthase (eNOS) expression in endothelia cells [17], increased expression of vascular endothelial growth factor (VEGF) in vascular smooth muscle cells, [18] and decreased NO production in smooth muscle cells [19]. Activation of PKC by hyperglycemia also induces expression of transforming growth factor *β* (TGF-*β*), which is suggested to be the major mediator of fibrosis in the diseases associated with sclerosis, such as diabetic nephropathy [20]. The PKC pathway is also implicated in NF-*κ*B activation, which connects hyperglycemia-induced oxidative stress to inflammation [21].

#### 2.1.3. Increased Intracellular Production of AGEs

AGEs are proteins, lipids, or nucleic acids that become nonenzymatically glycated or glycooxidated after exposure to aldose sugars. As a consequence of AGE accumulation, many proteins lose their function in vivo, including proteins involved in the regulation of gene transcription [22]. AGEs affect both extracellular function and intracellular function. In the extracellular matrix, accumulation of AGEs contributes to the formation of cross-links that permanently alter cellular structure [23]. AGEs that accumulate in the subendothelium induce monocyte migration across endothelium cell monolayer, which leads to activation of NF-*κ*B [24]. AGEs bind with one of their receptors, termed receptor for advanced glycation end products (RAGE) and exert their actions partly by influencing intracellular functions. Activation of NAPDH oxidase is speculated to be the primary mechanism by which AGEs-RAGE induces oxidative stress, which in turn transduces multiple signals, eventually resulting in NF-*κ*B activation, cytokine formation, and activation of proinflammatory pathways. AGEs are bound to RAGE in the endothelial cells, pericytes, and retinal pigment epithelial cells and initiate a wide range of cellular events that eventually lead to retinal damage in DR [25].

#### 2.1.4. Overactivation of the Hexosamine Pathway

Hyperglycemia-induced oxidative stress also contributes to the pathogenesis of diabetes and its complications by increasing the flux of fructose 6-phosphate into the hexosamine pathway. Fructose 6-phosphate is diverted from the glycolytic pathway. In the hexosamine pathway, fructose 6-phosphate acts as the substrate for the rate-limiting enzyme, glutamine:fructose 6-phosphate amidotransferase (GFAT), and is converted to glucosamine 6-phosphate, which is subsequently converted to uridine diphosphate-N-acetylglucosamine (UDPGlcNAc). UDPGlcNAc then attaches to the specific serine and threonine residues in transcription factors, leading to posttranslational modification of cytoplasmic and nuclear proteins. Inhibition of GFAT blocks hyperglycemia-induced increase in the transcription of transforming growth factor (TGF) *α*, TGF-*β*1 [26], and PAI-1 [27]. Hyperglycemia has been shown to induce increased activation of Sp1 through the hexosamine pathway. Sp1 activation then leads to the activation of the plasminogen activator inhibitor-1 (PAI-1) in vascular smooth muscle cells [28] and TGF-*β*1 and PAI-1 in arterial endothelial cells [27]. PAI-1 is upregulated by both the PKC and hexosamine pathways.

#### 2.1.5. Unifying Mechanism of Hyperglycemia-Induced Cellular Damage

In 2005, Brownlee [4] proposed a unifying mechanism that interconnects the hyperglycemia-induced processes, and, in the initial observation, hyperglycemia was proven to increase oxidative stress and ROS production related to diabetic damage. The hypothesis was based on evidence from cell culture and animal models, in which specific inhibition of various pathways ameliorate abnormalities induced by diabetes [19, 29, 30]. Clinical trials with pharmacologic agents that inhibited one of the specific pathways, for example, inhibition of AGE formation [13] or PKC activation [11, 12], showed disappointing results. Later, the unifying mechanism has been established to be the excess superoxide molecules produced by the mitochondrial electron transport chain linking hyperglycemia and oxidative stress, as well as the hyperglycemia-induced pathogenic mechanisms previously described [31].

During the course of diabetes, every cell in the patient is exposed to abnormally high glucose concentrations. However, high glucose-related damage only targets specific tissues, including retina, kidney, and nerve tissues. Cells in these tissues are deficient in the ability to change glucose transport rates when faced with elevated extracellular glucose concentrations, thereby leading to high intracellular glucose [32]. In cells with high intracellular glucose concentrations, higher amounts of glucose are metabolized and oxidized through the tricarboxylic acid (TCA) cycle, thereby increasing the flux of NADH and FADH2 into the mitochondrial electron transport chain and causing the accumulation of excess electrons to coenzyme Q, which eventually leads to superoxide generation [33]. The mitochondrial superoxide, in turn, can amplify the damage by activating other superoxide production pathways, including redox changes, Nox, and uncoupled eNOS. Manganese superoxide dismutase (MnSOD) is a mitochondrial superoxide-scavenging enzyme that has important functions in protecting cells from oxidative damage associated with exposure to ROS. Overexpression of MnSOD protects endothelial cells from hyperglycemia-induced oxidative damage, as well as the four pathological pathways previously discussed [31]. Later work confirmed that hyperglycemia-induced overproduction of mitochondrial superoxide activates the four pathological pathways by inhibiting GAPDH [4]. The inhibiting mechanisms act through the poly-ADP-ribose polymerase (PARP) pathway, which modifies GADPH through polymers of ADP-ribose. Hyperglycemia induces overproduction of ROS and DNA single-strand breaks, both of which can activate PARP, thereby resulting in modification of GADPH and reduction of its activity, and the process can be also prevented by MnSOD [34].

**Figure 1:**
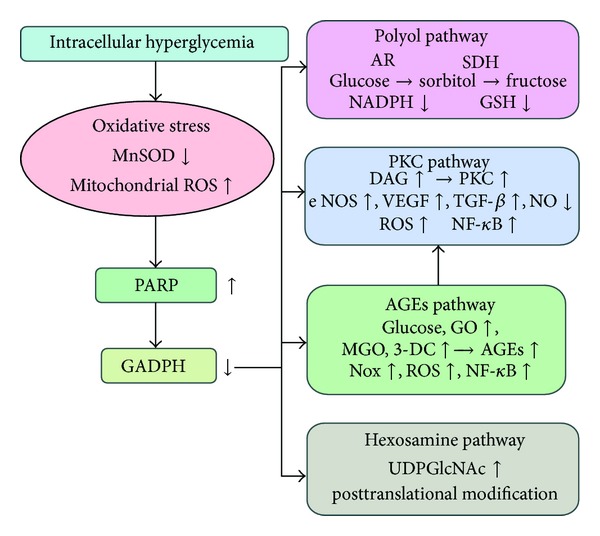
Relationship between hyperglycemia, oxidative stress, and pathways association with pathogenesis of diabetic retinopathy.

In summary, hyperglycemia induces mitochondrial generation of ROS, activates PARP, and reduces GAPDH activity, which in turn contributes to increased flux of the polyol pathway, activates PKC, increases intracellular production of AGEs, and overactivates the hexosamine pathway. This mechanism provides a refined link between hyperglycemia-induced oxidative stress and DR progression (Figure 1).

### 2.2. Hyperglycemic Memory and Oxidative Stress

In 1993, results of the initial DCCT study showed that intensive therapy reduces the occurrence and progression of diabetic complications, including DR [5]. The follow-up EDIC study began in 1994 [6]. In the study, patients who had been in the DCCT conventional therapy group adopted intensive therapy in the EDIC, and their level of glycemic control improved, as measured by the glycated hemoglobin (HbA1c) level. Meanwhile, the mean HbA1C level worsened in patients under the DCCT intensive therapy group. During the 14 years of EDIC follow-up, the HbA1c values for both groups became statistically identical [35]. Surprisingly, the results from EDIC showed that the patients in the conventional therapy group in the DCCT study continue to have a significantly higher incidence of complications even with improved glycemic control during the 14 years of EDIC. This phenomenon was described as “glycemic memory” [36]. Similar results were found from UKPDS and the posttrial monitoring program on type 2 diabetes patients. The intensive therapy group showed better glycemic control based on HbA1c content and had a significantly lower incidence of microvascular complications than the conventional therapy group. The phenomenon was termed “legacy effect” in the said publication [37]. Glycemic memory indicates several clinical significances. First, early intensive glycemic control is important. Second, the correlation of hyperglycemia as the only treatment may not prevent the subsequent development of diabetic complications. These significances lead to the third aspect, in which novel therapeutic agents that are potent in reversing glycemic memory may be needed.

Accumulating evidence has demonstrated that oxidative stress also contributes to glycemic memory in DR. Evidence from clinical studies on type 1 diabetic patients suggests that long-lasting periods of hyperglycemia induce increased levels of oxidative stress, as measured in plasma nitrotyrosine, and persistence of endothelial dysfunction [38]. Results from experimental studies also indicate that oxidative stress has a critical function in glycemic memory. Experimental glycemic memory animal model was induced in STZ-induced diabetes rats, with a period of poor glycemic control, followed by a period of good glycemic control, and failed to reverse oxidative modifications in retina [39]. Further study found that reversal of hyperglycemia fails to normalize peroxynitrite accumulation in the retina, and superoxide MnSOD remains inadequately scavenged [40]. Several in vitro experiments that used HUVESc [41], ARPE-19 retinal cells [42], and retinal pericytes (our unpublished data) show continued overproduction of ROS after glucose normalization.

**Figure 2:**
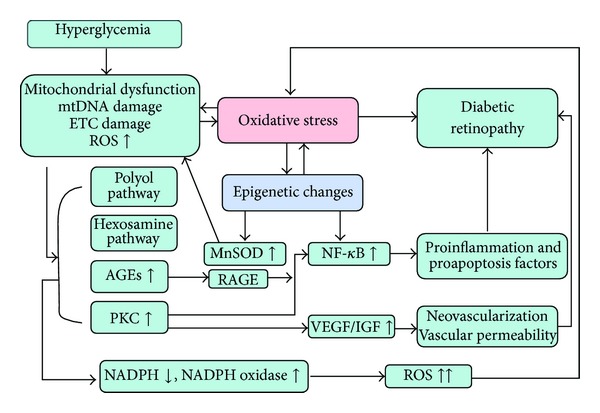
Oxidative stress is possibly a key mechanism in the hyperglycemia phenomenon.

The molecular mechanisms of oxidative stress that contribute to DR glycemic memory still remain to be fully elucidated, but many laboratories and researchers have already focused on mitochondrial abnormalities and epigenetic changes in related proteins. A series of studies that focused on mitochondria using experimental models of hyperglycemia memory demonstrated that temporarily poor glycemic control results in damage to mitochondrial structure and function [43], mitochondrial DNA (mtDNA) modulation and damage [44], impaired mtDNA repair machinery [44], and biogenesis [45]. These mitochondrial abnormalities persisted even after glucose stress is terminated. Mitochondrial damage was considered as the major cause of ROS overproduction, which induces epigenetic changes in various genes and transcription factors and consequently results in the deterioration of mitochondrial dysfunction and enhanced production of ROS. A recent experiment using STZ-induced diabetic rats and experimentally induced galactosemia models showed that glycemia memory induces histone modification at the retinal manganese superoxide dismutase gene (SOD2). The obtained data demonstrated that hyperglycemia increases trimethyl histone H4 lysine 20 (H4K20me3), acetyl histone H3 lysine 9 (H3K9), and NF-*κ*B p65 at the promoter and enhancer of retinal SOD2, resulting in gene-activating epigenetic changes in MnSOD and decreased expression of SOD2. Reinstitution of normal glycemia fails to resolve the observed abnormalities [46]. Zheng et al. [47] recently hypothesized that Class III histone deacetylase sirtuin 1 (SIRT1) has a major function in the phenomenon of hyperglycemia memory. The data provided showed that in both bovine retinal capillary endothelial cell (BREC) group and STZ-treated rats, NF-*κ*B p65 and the proapoptotic gene Bax are increased by hyperglycemia-induced ROS and remain elevated after normal glycemia was restored. The findings are consistent with the previously published data. Evidence provided that SIRT1 regulates apoptosis, inflammatory responses, and the levels of ROS. This finding is supported by the obtained data, in which downregulation of SIRT1 in BREC led to sustained overexpression of NF-*κ*B and Bax, whereas overexpression of SRT1 led to decreased expression of NF-*κ*B and Bax. These results partly explain the transient process of oxidative stress that induces long-term modifications and as an important molecular mechanism in the hyperglycemia memory in DR. (Figure 2).

### 2.3. MS, Type 2 Diabetes, and Oxidative Stress

MS is characterized by central obesity, dyslipidemia, hyperglycemia, and high blood pressure. Increasing evidence has shown that MS causes microvascular complications in patients with type 2 diabetes. Several clinical studies indicate that patients with MS have a high prevalence of retinopathy [48]. Data show that people without diabetes also develop retinopathy, suggesting that factors other than hyperglycemia are also associated with retinal lesions. The results are obtained from two large-scale prospective studies, namely, the UKPDS [7] and Steno-2 study [8]. Both studies suggest that besides glycemic control, treatment of hypertension and dyslipidemia may be even more important for the prevention of both microvascular and macrovascular diabetic complications. The association between diabetes and hypertension with retinopathy is well known [49]. Recent several studies have focused on obesity and dyslipidemia with DR. The Hoorn study reported in 2002 [50] indicated that hyperglycemia and retinopathy are also associated with blood pressure, lipid concentration, and body mass index. The Handan Eye Study [51] and the Inter99 Eye Study [52] also showed similar results. Results from the ADVANCE study [53] failed to show an association between low HDL cholesterol (HDL-C) and retinal lesions but suggested that HDL-C level is an independent risk factor for the development of nephropathy, another diabetic microvascular complication. These clinical results demonstrate that the risk factors for retinopathy in subjects with MS and type 2 diabetes differ from the risk factors in subjects with type 1 diabetes.

Recent studies have focused on whether oxidative stress and mitochondrial dysfunction are contributory factors for cellular and tissue damage in MS and type 2 diabetes. Both MS and type 2 diabetes are characterized by disturbances in fatty acid metabolism and accompanied by the accumulation of free fatty acids (FFAs) in nonadipose tissues. A large proportion of free FFAs delivered from lipolysis in the mitochondria are attributed to the disorder in mitochondrial fuel metabolism, which is characterized by excessive *β*-oxidation, impaired switching to carbohydrate substrate, and decreased TCA cycle activity. This phenomenon results in incomplete oxidized products [54] that cause increased production of superoxide through the mitochondrial electron transport chain. Both humans and rodents with high dietary fat intake exhibit overproduction of superoxide in the mitochondria of skeletal muscle fibers [54]. The phenomenon further suggests mitochondrial overload as a direct mechanism by which excessive lipid supply leads to oxidative stress damage in MS and T2D. Increased oxidation of intracellular fatty acids also leads to increased mitochondrial NADH/NAD+ ratio and results in activation of the same mechanisms as hyperglycemia-induced ROS, including PKC, AGEs, and NF-*κ*B [55].

**Figure 3:**
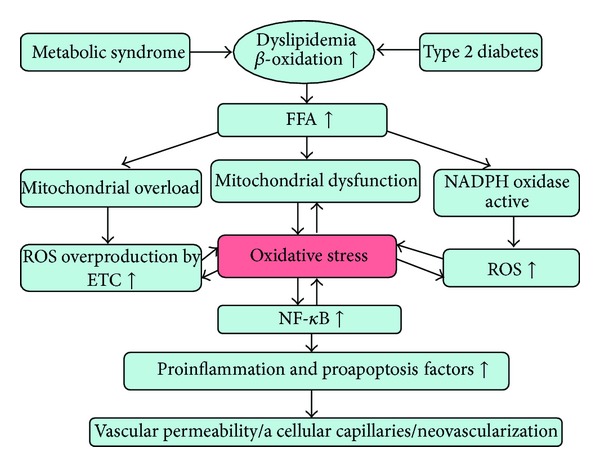
Oxidative stress also plays a pivotal role in dyslipidemia induced retinal damage.

Several other mechanisms are implicated in lipid-induced oxidative stress in DR, including nonmitochondrial ROS production by Nox. A morphological study in BBZ/Wor rats, an obese, noninsulin dependent model of diabetes, first demonstrated increased Nox activity and endothelial cell dysfunction in retina [56]. In saturated FFA palmitate-cultured bovine retinal pericytes, apoptosis in cells is associated with activation of Nox and signaling through the NF-*κ*B pathway [57]. In experimental cell studies that use human retinal pericytes, highly oxidized-glycated low-density lipoprotein (HOG-LDL) can induce apoptosis of those cells [58]. A subsequent study that used human retinal Müller cells also suggested that HOG-LDL may be implicated in apoptotic Müller cell death. Increased Nox 4 was found in the model, and either N-acetyl-cysteine (NAC, a blocker of oxidative stress) or 4-phenylbutyrate (a blocker of endoplasmic reticulum stress) partly attenuated apoptosis [59]. Loss of pericytes results in endothelial cell proliferation and enhances abnormal angiogenesis in the retina, and the death or dysfunction of Müller cells contributes to the breakdown of the blood-retina barrier (BRB), both of which are cardinal features of early DR. In the db/db mice model, expression levels of Nox 4 and VEGF are significantly increased in the retinas and reduced by lovastatin, a drug used for the treatment of dyslipidemia. An in vitro study used retinal endothelial cells and suggested that hypoxia and high glucose induce activation of Nox 4 associated with ROS generation, VEGF expression, and BRB breakdown. The protective effect of lovastatin is partly through inhibition of Nox 4 activation [60]. Further in vivo studies are needed, but the aforementioned evidence suggests that Nox activation is implicated in the pathogenesis of DR in MS and type 2 diabetes (Figure 3).

Overactivation of the renin-angiotensin system (RAS) has a critical function in both MS and type 2 diabetes [61]. Angiotensin (ANG) II, a distal element of RAS, can activate vascular Nox by PKC and c-Src dependent pathways [62], thereby resulting in the production of superoxide ions via a one-electron reduction of oxygen and the oxidation of NADPH. In type 2 diabetic rat models, researchers observed significantly increased angiotensin-converting enzyme levels, as well as increased levels of VEGF and p22phox, an Nox subunit, which can be significantly reduced by candesartan, an ANG II receptor blocker (ARB) [63]. Compared with the mice model induced with diabetes only, concomitance of hypertension and diabetes in the other mice model exacerbates oxidative stress, neurodegeneration, and mitochondrial dysfunction in the retina. ARB treatment with losartan reestablishes all of the aforementioned parameters. The study first indicated ARB as a potential treatment for DR by reestablishing oxidative redox [64]. Increasing evidence has shown that ARB attenuates retinal damage in diabetes. The protective effect of ARB may be due, in part, to reduced production of Nox-derived ROS.

The pathogenesis of DR in MS and type 2 diabetes is not clearly elucidated, partly because an appropriate model has not been developed. However, oxidative stress and mitochondrial dysfunction have been proven to be vitally important in the progression of DR.

## 3. Antioxidant Therapy in DR

DR management requires an individualized treatment with combined multidisciplinary approach. Laser photocoagulation remains one of the most currently used retinopathy management strategies. Other strategies include intravitreal injection of VEGF agents or steroids and surgical management. Systemic medical management includes optimization of glycemic control, blood pressure, and lipid metabolism [65]. Both clinical trials and experimental results provide rational basis for therapeutic interventions to assess whether the balance between oxidant and antioxidant can induce or slow the progression of the diseases. Therefore, antioxidative stress therapy may also be implicated in the therapeutic strategy of DR.

### 3.1. Antioxidant Supplementation

Supplementation with novel antioxidants is the first attempt in developing therapeutic strategies for DA. Screening and review of all the antioxidants used for treatment in experimental and clinical DR cannot be carried out, but the following antioxidants have already been studied extensively: vitamins C and E,* alpha*-lipoic acid, NAC,* beta*-carotene, and taurine. Several studies show that primary antioxidants or genetic manipulation of antioxidant defenses can at least partially ameliorate oxidative stress and consequently reduce severity of DR in cell culture and animal models [66–80] (Table 1).

Despite the remarkable protective effects of antioxidant supplementation on experimental studies, clinical studies show conflicting results [81–84]. High dose use of some antioxidants can result in some serious side effects [67]. Discrepancies in the results between experimental and clinical trials may be due to the limitations of clinical trials, such as the given dose, concomitant disease, other medication, course and severity of the disease, gene, and environmental factors. Moreover, ROS also functions in key signaling pathways, which are essential for proper cell function. For example, at physiological concentrations, ROS activate receptor tyrosine kinases and transcriptional factors, such as NF-E2-related factor 2, which then binds to the antioxidant response element (ARE) and leads to expression of antioxidants [85]. Direct scavenging of ROS by antioxidant supplementation can also interfere with signaling pathways, which can partially explain why antioxidant supplementation failed to demonstrate effectiveness in the majority of clinical trials. Nevertheless, antioxidant supplementation should still be considered, but other medications and target patients should be further explored. Most importantly, efforts must be focused on understanding the mechanism underlying oxidative stress and exploring new drugs that interfere with the cause of oxidative stress.

### 3.2. Therapies Based on the Mechanisms of Oxidative Stress

Considering that existing treatments for diabetes do not prevent DR, new drugs and strategies have been explored based on targeting oxidative stress, inflammatory pathways, and hyperglycemia-induced metabolic stress pathways. Oxidative stress-related pathways and their inhibition are reviewed in this section.

#### 3.2.1. PARP Inhibitors

PPAR activated in retina increases oxidative stress and promotes DNA damage by inhibiting the activity of GAPDH, which then increases the flux into the four pathways induced by hyperglycemia. The specific PPAR inhibitor PJ-34 was found to significantly inhibit diabetes-induced apoptosis of retinal microvascular cells and the development of early lesions of DR in diabetic rats. PJ-34 inhibits NF-*κ*B activation and induction of the expression of inflammatory proteins by NF-*κ*B [86]. In STZ-diabetic rats treated with the PPAR inhibitors 1,5-isoquinolinediol (ISO) and 10-(4-methyl-piperazin-1-ylmethyl)-2H-7-oxa-1,2-diaza-benzo-[de]an thracen-3-1 (GPI-15427) for 10 weeks, retinal oxidative-nitrosative and endoplasmic reticulum stress and glial activation are significantly reduced compared with diabetic rats without PPAR inhibitor treatment [87]. The diabetes-induced upregulation of PARP, ROS, ERK1/2 phosphorylation, and cleaved caspase-3 and downregulation of brain-derived neurotrophic factor, synaptophysin, and glutamine synthetase in the retinas of diabetic rats are prevented by ISO [88]. These findings suggest a beneficial effect of PPAR inhibitors in the development of DR and demonstrate that inhibition of PPAR is a promising therapeutic target.

#### 3.2.2. Transketolase Activators

Transketolase activators, including thiamine and benfotiamine, have been recently considered as potential therapeutic agents. When increased superoxide inhibits GAPDH activity, glycolytic intermediates above the enzyme accumulate. Among the glycolytic intermediates, fructose 6-phosphate and glyceraldehyde 3-phosphate are also final products of the transketolase reaction. Transketolase can reduce the concentration of these two glycolytic intermediates and thereby inhibit their flux into the damage-inducing pathways activated by hyperglycemia. The procedure requires the vitamin thiamine as a cofactor. The thiamine derivative benfotiamine is an effective transketolase activator. Benfotiamine treatment prevents acellular capillaries in the retina of diabetic rats by blocking the major pathways involved in hyperglycemia, including AGEs, PKC, and hexosamine pathways [89]. Diabetes has recently been found to cause intracellular thiamine depletion, which provides further evidence that thiamine and benfotiamine are potential therapeutic agents [90]. Recently, experimental studies that used bovine retinal endothelial cells have showed that the mechanism by which high glucose inactivates GAPDH in the retina is through nitrosylation and ribosylation of GAPDH. Inhibiting covalent modification of GAPDH can have immense clinical significance as a potential target [91].

#### 3.2.3. NF-*κ*B Inhibitors

Hyperglycemia-induced PKC and AGE pathways are both associated with NF-*κ*B activation and expression of downstream gene targets that are involved in the pathogenesis of DR. Moreover, lipid-induced activation of Nox activates several signaling pathways, with NF-*κ*B as a central molecule. Thus, several studies assess the use of NF-*κ*B inhibitors in the treatment of experimental diabetic models. Two NF-*κ*B inhibitors, namely, dehydroxymethylepoxyquinomicin (DHMEQ) and pyrrolidinedithiocarbamate, have been used in diabetic rats. Nagai et al. [92] showed that DHMEQ attenuates the retinal expression of ANG II, VEGF, and other inflammatory proteins in diabetic rats. Another NF-*κ*B inhibitor, pyrrolidinedithiocarbamate, suppresses ischemia-induced retinal neovascularization [93]. Therefore, NF-*κ*B inhibitors can be considered another class of potential agents for preventing and controlling DR.

### 3.3. Multitarget Drugs

Both clinical and experimental evidence suggest that reducing DR requires systemic management, including early intensive glycemic control, antihypertension, and antihyperlipidemia treatments. Based on this approach, drugs with multiple targets can prove highly useful in the pharmacology of DR.

#### 3.3.1. Fenofibrate

Evidence suggests that dyslipidemia and serum fatty acids are associated with increased oxidative stress and other abnormalities in the retina, leading to the progression of DR. Some lipid-lowering drugs have great potential in preventing retinal damage and include fenofibrate and statins. Both the Fenofibrate Intervention and Event Lowering in Diabetes and Early Treatment Diabetic Retinopathy Study show robust and consistent clinical data to recommend fenofibrate as an adjunctive treatment for DR [94]. In the Action to Control Cardiovascular Risk in Diabetes Eye study, the combination of fenofibrate and simvastatin reduces the rate of progression of DR by 40% compared with simvastatin alone [95]. Conversely, reduced progression of retinopathy is not observed in two large-scale studies of statins [96]. The proposed mechanisms implicated in the mode of action of fenofibrate involve lipid and nonlipid pathways, including protective effects on oxidative stress, apoptosis, inflammation, BRB breakdown, and neuroprotection [97]. Thus, fenofibrate may be considered as a preventive therapeutic strategy for patients with DR. Elucidation of the mechanisms of fenofibrate is indicated and will further help explore different administration strategies and target groups.

#### 3.3.2. ARB

A local RAS is identified in the eye and upregulated in active retinopathy, suggesting the involvement of RAS in the pathogenesis of DR [98]. Elucidation of the function of RAS blockade in the new approaches to retinopathy prevention receives considerable attention. ARB therapy can slow down the progression of diabetic nephropathy [99]. In addition, growing evidence suggests that ARB may also have a preventive effect, over and above their blood pressure lowering effect, in the pathogenesis of DR.

Candesartan is an angiotensin II type 1 receptor antagonist that inhibits the actions of angiotensin II on the RAS. Candesartan was introduced in 1998 for the treatment of hypertension. Recently published data have suggested the usefulness of candesartan drug in the treatment of stroke, heart failure, diabetic nephropathy, and most recently in preventing the progression of DR [100]. The Diabetic Retinopathy Candesartan Trials (DIRECT) was set up to assess use of candesartan in subjects with type 1 [101] and type 2 diabetes [102]. The primary trial end-points are not met, but candesartan clearly reduces severe retinopathy. The mechanism for the preventive effect of candesartan has not been examined in the DIRECT program and may be partially beyond the effect of blood pressure lowering because the observed beneficial effects remained significant after adjustment for blood pressure. Current understanding of how candesartan inhibits the development of DR is via reduction of the accumulation of AGEs and overexpression of VEGF [103].

Other members of the ARB family, such as losartan [104] and valsartan [105], also have beneficial effects in the treatment of DR. Thus, ARBs may be considered as one of the most potentially useful agents for ameliorating in the DR.

## 4. Conclusion

Oxidative stress has a very important function in the pathogenesis of DR in both type 1 and type 2 diabetes. In the present review, the pathogenesis of this diabetic complication is discussed in detail, with special focus on oxidative stress. Current and potential therapies associated with oxidative stress are also introduced.

Oxidative stress has been implicated as a contributor to both the onset and the progression of DR. Among numerous molecular mechanisms identified in DR, oxidative stress may be the instigator and the most common etiologic factor.

DR remains the cornerstone of management, and the vision prognosis of the surgery is poor. All the clinical therapies aim to treat the abnormal retinal vasculature at advanced stages of DR, and several physiological and functional abnormalities, such as neuroretinal dysfunction, develop before this histopathology appears. Identifying the most effective methods of treatment and drugs for preventing retinopathy or intervening at an early, asymptomatic stage is necessary to preserve vision. Regarding the functions of oxidative stress and factors that affect the pathogenesis of DR, therapeutic correction of oxidant-antioxidant balance may be a powerful approach for preventing vision loss associated with DR. While the lack of clinical evidence on the beneficial effects of conventional antioxidant in DR should not hinder us from further and deeper research on the area, clinical practice should be based on the results of numerous clinical trials. In most of the clinical trials, antioxidants were administered systemically. However, administering effective drug concentrations in the retina is difficult to achieve because the blood-retina barrier (BRB) limits drug permeation. Local application represents an effective and safe methodology because side effects associated with systemic administration can be reduced. Thus, development of new drug delivery systems for topical use of antioxidants in ocular is a promising therapeutic strategy. Development of new drugs in the prevention and treatment of DR still faces great challenges and requires further investigation through experimental and clinical studies.

## Figures and Tables

**Table 1:** Antioxidants used in the treatment of DR and their mechanisms of action.

Antioxidants	Mechanisms	References
Vitamins C and E	Free radical scavenging; prevented the inhibition of retinal glutathione reductase, glutathione peroxidase (GPx), and SOD activities	[66, 81, 83]

Vitamin E	Radical scavenger, against lipid peroxidation	[67]

Ascorbic acid, acetate,	Decreased retinal oxidative stress, protein kinase C activity, and nitric oxides; decreased retinal cell loss	[66, 82, 84]
and *α*-tocopherol		

*beta*-Carotene	High tendency to oxidize; decreased retinal oxidative stress, protein kinase C activity, and nitric oxides; decreased retinal cell loss	[66, 83]

*alpha*-Lipoic acid	Inhibited activation of NF-*κ*B and cell apoptosis; decreased retinal oxidative stress; prevented the reduction of GSH and GPx levels; reduced malondialdehyde (MDA) levels; decreased expression of angiogenic factors	[68–71, 82]

N-Acetylcysteine (NAC)	Inhibition activation of NF-*κ*B and cell apoptosis; decreased ROS. VEGF and ICAM-1 expression; inhibited activation of macrophage/microglia; inhibition of perivascular cell changes	[68, 72, 73]

Aminoguanidine	Prevented elevation of retinal oxidative stress, NO, and PKC activity; inhibited lipid peroxidation and AGEs formation	[74, 75]

Curcumin	Multipotent activities prevent the decrease in the antioxidant capacity and increase in oxidative stress; inhibit diabetes-induced elevation in the levels of IL-1beta, VEGF, and NF-*κ*B	[76]

Pycnogenol	Free radical scavenging; anti-inflammatory and capillary protective activities	[77]

Taurine	Multipotent activities prevent the changes in ultrastructure of retina and apoptosis in retinal glial cells; increasing activities of antioxidant enzymes and intracellular GSH	[78, 79]

Zinc	Reversed the depleting effect on retinal GSH	[80]
